# Loss of Ep-CAM (CO17-1A) expression predicts survival in patients with gastric cancer

**DOI:** 10.1038/sj.bjc.6602519

**Published:** 2005-05-03

**Authors:** I Songun, S V Litvinov, C J H van de Velde, S T Pals, J Hermans, J H J M van Krieken

**Affiliations:** 1 Department of Surgery, Leiden University Medical Center, PO Box 9600, 2300 RC Leiden, The Netherlands; 2 Department of Pathology, Leiden University Medical Center, PO Box 9600, 2300 RC Leiden, The Netherlands; 3 Department of Pathology, Academic Medical Center, PO Box 22660, 1100 DD Amsterdam, The Netherlands; 4 Department of Medical Statistics, Leiden University Medical Center, PO Box 9600, 2300 RC Leiden, The Netherlands; 5 Department of Pathology, Radbond University, Nijmegen Medical Center, PO Box 9101, 6500 HB Nijimegen, The Netherlands

**Keywords:** Ep-CAM, CD44, immunohistochemistry, prognostic factor, survival, gastric cancer

## Abstract

Preoperative staging of gastric cancer is difficult and not optimal. The TNM stage is an important prognostic factor, but it can only be assessed reliably after surgery. Therefore, there is need for additional, reliable prognostic factors that can be determined preoperatively in order to select patients who might benefit from (neo) adjuvant treatment. Expression of immunohistochemical markers was demonstrated to be associated with tumour progression and metastasis. The expression of p53, CD44 (splice variants v5, v6 and v9), E-cadherin, Ep-CAM (CO17-1A antigen) and c-erB2/*neu* were investigated in tumour tissues of 300 patients from the Dutch Gastric Cancer Trial, investigating the value of extended lymphadenectomy compared to that of limited lymphadenectomy). The expression of tumour markers was analysed with respect to patient survival. Patients without loss of Ep-CAM-expression of tumour cells (19%) had a significantly better 10-year survival (*P*<0.0001) compared to patients with any loss: 42% (s.e.=7%) *vs* 22% (s.e.=3%). Patients with CD44v6 (VFF18) expression in more than 25% of the tumour cells (69% of the patients) also had a significantly better survival (*P*=0.01) compared to patients with expression in less than 25% of the tumour cells: 10 year survival rate of 29% (s.e.=3%) *vs* 19% (s.e.=4%). The prognostic value of both markers was stronger in stages I and II, and independent of the TNM stage. Ep-CAM and CD44v6-expression provides prognostic information additional to the TNM stage. Loss of Ep-CAM-expression identifies aggressive tumours especially in patients with stage I and II disease. This information may be helpful in selecting patients suitable for surgery or for additional treatment pre- or postoperatively.

Patients with gastric carcinoma have a poor prognosis, especially the patients with an advanced stage disease (Allum *et al*, 1989; Akoh *et al*, 1991; Wanebo *et al*, 1993). In TNM stages I and II, better survival rates are seen after a curative resection: 5 year survival rates of 83–99% for stage I and 48–70% in stage II (Miwa, Japanese Research Society for Gastric Cancer, 1984; Hermanek, 1985). The TNM stage thus is an important prognostic factor, but it can be assessed reliably only after surgery and is therefore of no use for patient selection before surgery. Furthermore, within stages I and II, a significant number of patients will suffer from recurrent disease. Especially within this group there is a need for additional prognostic factors that can be determined preoperatively. Such factors are potentially helpful in identifying patients that might benefit from additional therapeutic modalities, either pre- or post-operatively (Yu *et al*, 1998; Hermans *et al*, 1999).

The process of carcinogenesis and metastasis is complex. Since we were interested in factors, which can predict whether the tumour is beyond cure by surgery alone, we reasoned that especially molecules involved in cell–cell and cell–extracellular matrix (ECM) interactions might be of high relevance. CD44, a hyaluronate receptor, is involved in cell migration through the ECM, which can be viewed as highly relevant for tumour invasion and metastasis. Previous studies have indicated that CD44 isoform expression may be related to gastric tumour progression and poor prognosis for the patient (Heider *et al*, 1993; Mayer *et al*, 1993; Mulder *et al*, 1994; Terpe *et al*, 1994; Günthert *et al*, 1995). Given the fact that progression of tumours is modulated by changes in cell–cell interactions of the progressing tumour clones, we especially focused on the expression of Ep-CAM, an epithelial cell adhesion molecule, involved in regulation of cadherin adhesions, and, possibly, cell proliferation and invasion (Litvinov *et al*, 1994a, 1994b; Litvinov, 1995). Furthermore, we analysed whether the expression of two molecules often involved in gastric cancer, p53 (involved in cell cycle control and apoptosis, and often overexpressed when mutations are present) and *neu* (or erbB2, a receptor tyrosine kinase that can be overexpressed due to the gene amplification, Falck and Gullick, 1989; Houldsworth *et al*, 1990) might be associated with prognosis in gastric cancer, even though they are presumably associated with early events in gastric carcinogenesis.

Evaluation of prognostic factors has to be based on high quality clinical and pathological data. In the Netherlands, a prospectively randomised, multicenter trial was conducted to compare the therapeutic efficacy of *extended* lymph node dissection with *limited* lymph node dissection in patients with gastric cancer (Bonenkamp *et al*, 1995, 1999). Strict quality control measures were taken to obtain optimal lymph node retrieval and thus postoperative staging. The prospectively collected clinical and pathology data from this trial form an optimal basis to evaluate the usefulness of prognostic value of immunohistochemically determined protein expression in tumour cells.

## MATERIAL AND METHODS

### Patient selection

Between August 1989 and July 1993 a prospective randomised multicenter trial was conducted in the Netherlands (Dutch Gastric Cancer Trial, DGCT) to compare the therapeutic efficacy of *extended* lymph node dissection (N1 and N2 levels, so-called D2) with that of *limited* lymph node dissection (N1 level, so-called D1) in patients with gastric cancer, operated on with curative intent (R0). For these patients (criteria for curative resectability were published earlier, Bonenkamp *et al*, 1995, 1999), presence of nodal involvement was assessed histologically and the pathologist recorded the actual number and location of the lymph nodes retrieved. Follow-up of the patients is at least 10 years.

In the present study, tumour tissue of 300 patients was used. Selection of these patients was based on the hospitals entering the largest number of patients, in order to have minimal variability in preparation and preservation of patient material used in this study.

### Immunohistochemistry

In order to evaluate protein expression in adenocarcinoma of the stomach, formalin-fixed, paraffin-embedded tissue blocks of the primary tumour were used. According to the allocated treatment, the specimens were obtained by D1 or D2 resection. If a curative resection in intent was not possible, a palliative procedure was performed. From each resection specimen one tissue block was selected that contained the largest amount of tumour.

Sections (4-*μ*m thick) were cut from formalin-fixed, paraffin-embedded tissue blocks, mounted on precoated slides and kept at 37°C overnight. All paraffin sections were dewaxed in xylol for 20 min and endogenous peroxidase activity was blocked by methanol/H_2_O_2_.

The monoclonal antibodies of the following specificities were used: cell cycle regulator p53 (mAb NCL-p53-DO7, Novocastra Laboratories Ltd), Ep-CAM (mAb 323/A3, Centocor, Malvern, PA, USA), E-Cadherin (mAb HECD-1, Thamer Diagnostica B.V.), CD44, splice variant v5 (VFF8), v6 (VFF7 and VFF18)^9^, and v9 (all from Bender Co., Vienna, Austria) and *neu* (Department of Pathology, Leiden). In negative controls, the primary antibody was replaced by phosphate-buffered saline (PBS).

For Ep-CAM staining, the sections were pretreated with a trypsin-solution (0.1% trypsin with 0.1% CaCl_2_), pH 7.4, at 37°C for 20 min. For p53, E-cadherin, CD44 variants and *neu* staining, the sections were pretreated by microwave in citrate buffer (pH 6.0) for 25 min. The pretreated sections were rinsed in PBS and blocked by normal goat serum to reduce nonspecific antibody binding. The primary antibody was then applied and incubated overnight in its optimal dilutions in PBS/1% bovine serum albumin (BSA).

The sections were washed with PBS prior to incubation with the secondary antibody. Then, a double step detection system was used: biotinylated rabbit anti-mouse (RAM) IgG was followed by streptavidin-biotin-complex^HRP^ (sABC); each incubation was for 45 min. The slides were stained with 3,3′-diaminobenzidine/H_2_O_2_ (DAB) solution, and counterstained with Mayer's haematoxylin.

### Scoring of the sections

The expression of the proteins was scored according to the estimated percentage tumour cells in the total tissue section showing positive staining. For each marker a scoring system was developed after initial screening of the variation of expression of each marker and taking systems used in the literature in account. This resulted in the following categories: p53 (0: 0–10%; 1: ⩾10–100%); VFF8, VFF7 and NEU (0: negative; 1: positive); CD44v9 (0: 0–5%; 1: ⩾5–100%); VFF18 (0: 0–25%; 1: ⩾25–100%); E-Cadherin (0: <50%; 1: ⩾50%) and Ep-CAM (0: negative; 1: 1–99%; 2: 100%).

The scoring was done by two independent observers (IS and JHJMvK). Discrepancies were solved and consensus was reached by using a double-headed microscope. Incidentally, the marker scorings could not be performed because of missing material. Clinical data were provided after obtaining immunohistochemical results.

### Statistical analysis

For statistical analysis the SPSS program was used. Kaplan–Meier survival curves are compared using the log-rank test. Cox's regression was used to study the prognostic value for survival of tumour marker combinations. Differences were considered statistically significant, when the *P*-value was less than 0.05.

## RESULTS

The study group consisted of 181 male and 119 female patients. Mean age was 64.7 years (range 31–84). Of these patients, 154 received a D1 and 146 D2 lymphadenectomy; 254 patients (85%) had a resection with curative intent and the remaining 46 patients (15%) had noncurative procedures. As expected, the TNM stage (not always available in noncurative procedures) was highly prognostic (see Table 1).

Based on univariate analyses of the original scoring of the markers, all marker expressions were dichotomised into more or less equal groups. Only Ep-CAM is divided into three groups: 1, negative; 2, any loss of expression (1–99% positive) and 3, no loss of expression (100% positive). The results are reported in Table 1.

**Figure 1:**
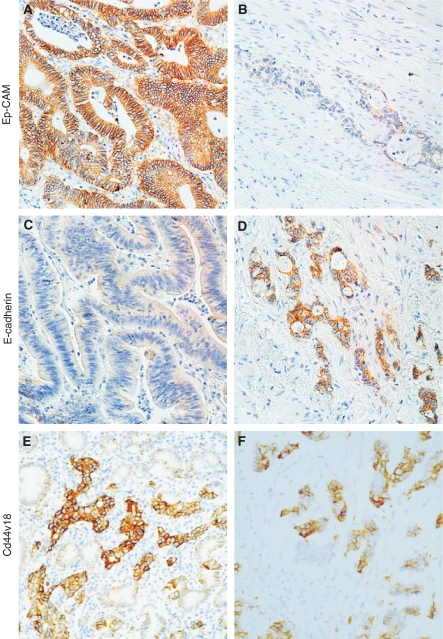
Examples of staining for CD44v6 (VFF18), E-Cadherin and Ep-CAM. Case 1: all cells are staining for Ep-CAM (**A**), no staining for E-Cadherin (**C**) and partial loss for CD44VFF18 (**E**). Case 2 shows only little staining for Ep-CAM (**B**), staining in more than 90% of the tumour cells for E-Cadherin, (**D**) whereas there is also more than 90% staining for CD44vvf18 (**F**).

Examples of the staining patterns for E-cadherin and CD44v6 are shown in Figure 1.

**Figure 2:**
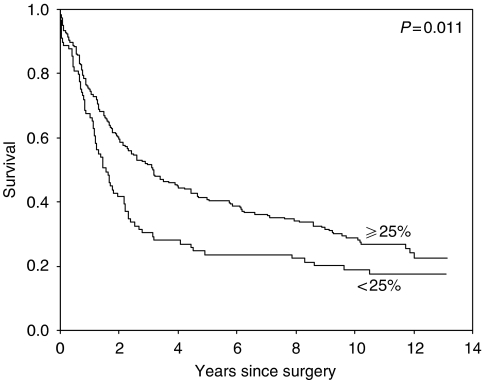
Survival of patients in whom more than 25% of the tumour cells stain for CD44v6 (VFF18) compared to those with less staining.

**Figure 3:**
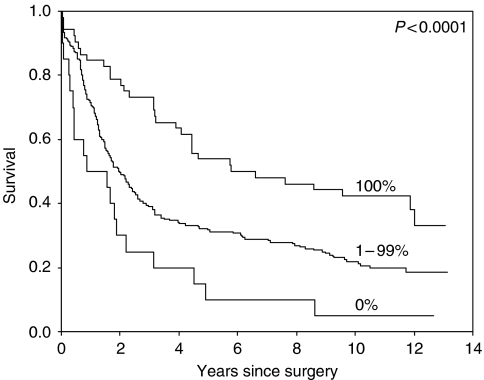
Survival curves of the three groups of patients with no staining for Ep-CAM compared to those with no loss and the intermediate group.

From all the markers tested, only CD44v6 (VFF18 antibody) and Ep-CAM had a statistically significant prognostic value for survival (see Table 1 and Figures 2 and 3).

As a curative resection in intent is an important prognostic factor itself, we analysed this group separately. Again, only the TNM stage, CD44v6 (VFF18) and Ep-CAM had a strong prognostic value (Table 2).

The prognostic value of the markers was studied additional to the TNM stage with a stepwise Cox's regression analysis. For this analysis the TNM stage was dichotomised into stages I+II *vs* stages III+IV. Both CD44v6 (VFF18) and Ep-CAM were selected as having a significant prognostic value additional to the TNM stage (Table 3).

**Figure 4:**
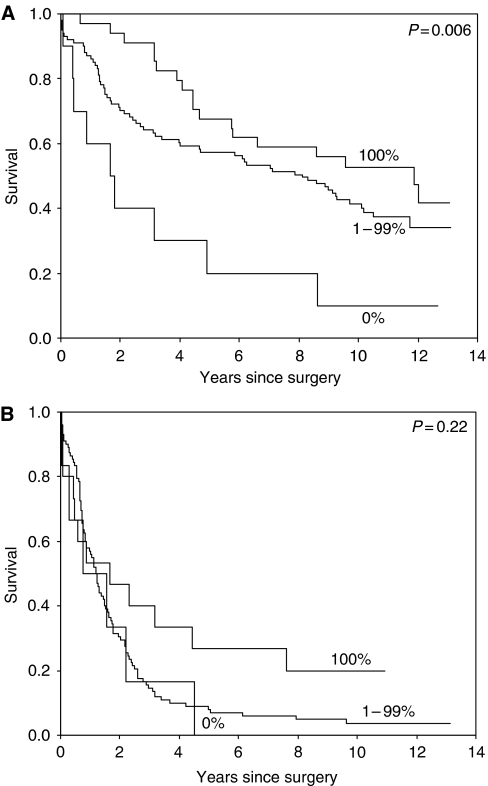
(**A**) Survival curve of the three groups divided by Ep-CAM staining for stages I+II patients only, showing that even patients with low stage disease, but with complete loss op Ep-CAM have very poor prognosis. (**B**) Survival curve of the three groups divided by Ep-CAM staining for stages III+IV patients only, showing no significant differences.

**Figure 5:**
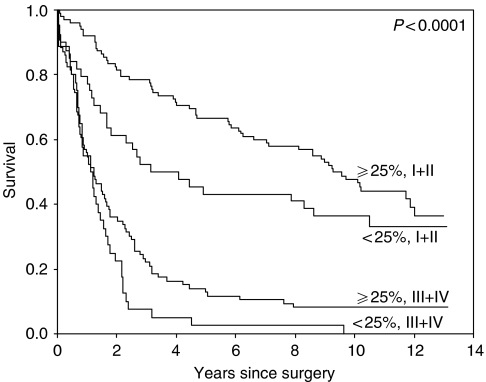
Survival curve of the three groups divided by Ep-CAM staining for stages III+IV patients only. Survival of patients by stage divided in those with less than 25% tumour cells staining for CD44v6 (VFF18) compared to those with more than 25% of the tumour cells staining, showing that the prognostic impact is independent of stage.

The prognostic value of the markers is especially evident in the stages I and II, see Figure 4A. In stages III and IV, the survival rates are very poor, so Ep-CAM expression cannot reach a statistically significant difference between the groups, see Figure 4B. Also, CD44v6 (VFF18) expression has prognostic relevance especially in early stages, see Figure 5.

## DISCUSSION

Our study demonstrates that loss of Ep-CAM and CD44v6 (VFF18) expression in gastric cancer has additional prognostic value to the TNM stage. If confirmed, these findings can be helpful in selecting patients for (neo-)adjuvant therapy for gastric cancer (Yu *et al*, 1998; Hermans *et al*, 1999).

Although Ep-CAM is present in many epithelial cell types, some, like squamous epithelia express this molecule only in embryogenesis or in neoplasia (reviewed by Litvinov, 1995). In gastric epithelium the expression of Ep-CAM is low, and its increase is associated with very early stages of development of intestinal metaplasia (unpublished data). However, Ep-CAM loss associated with poor prognosis, as demonstrated in our earlier (Songun *et al*, 1996) and the present study, differs from previous observations of increased Ep-CAM expression associated with poor prognosis in breast cancer (Mirecka *et al*, 1995). Although not very strong, Ep-CAM mediated adhesions were able to suppress the scattering of cells embedded into matrigel (Litvinov, 1995). It was also demonstrated that in carcinoma cells with low levels of E-cadherin, a role for Ep-CAM adhesions in interconnecting cells is increasing (Tandon *et al*, 1990; Basak *et al*, 1998). Basak *et al* (1998) showed that in a model system Ep-CAM mediated adhesion can suppress invasion of tumour cells grafted in mice. Therefore, it seems quite possible that Ep-CAM negative cells are greatly reduced in means of cell–cell adhesion, which promotes their metastasis. However, we did not find a prognostic impact for E-cadherin expression, in concordance with findings in colorectal cancer (Van der Wurff *et al*, 1992, 1997). No studies have been carried out on gastric cancer previously, except for hereditary diffuse gastric cancer, which is extremely rare (Suriano *et al*, 2003).

CD44 is a highly glycosylated cell surface molecule, which is involved in cell–cell and cell–matrix interactions (Haynes *et al*, 1989, 1991). It was proven that transfection with cDNA encoding one isoform of CD44 converted nonmetastatic carcinoma and sarcoma rat cells into metastatic cells (Günthert *et al*, 1991). In human, high CD44 expression was shown to correlate with tumour dissemination and poor prognosis in diffuse large cell lymphoma and in colorectal carcinoma (Koopman *et al*, 1993; Mulder *et al*, 1994). CD44 variants containing v6 were also upregulated in activated lymphocytes (Koopman *et al*, 1993). However, it was also reported that CD44v6 is downregulated in tumours of squamocellular origin. Also, better differentiated carcinomas displayed more intense reactivity than more undifferentiated ones (Salmi *et al*, 1993). Our data are in line with previous studies (Mirecka *et al*, 1995; Muller *et al*, 1997). Both in the latter studies and in our study there was no association between CD44v6 expression (with VFF18) and presence of lymph node metastasis or tumour stage (data not shown). Our results suggest that CD44v6 would not be responsible for invasive growth and metastasis formation. They rather suggest that CD44v6 containing tumours behave less aggressively, illustrated by the fact that they are associated with significantly better survival. However, the VFF7 antibody, which is less sensitive in detecting CD44v6, was staining far fewer tumour cells and its expression was less strong compared to the VFF18 antibody. This shows the importance of selection of the right antibodies in studies like the present one.

The large amount of studies on p53 as prognostic marker gives very variable results. Our study, using just immunohistochemistry, the only generally applicable method, does not show clinical relevance. Our findings suggest the following biological implications: loss of p53 protein or E-cadherin is an early event during oncogenesis and therefore not predictive of a metastatic behaviour (Van der Wurff *et al*, 1992), whereas CD44 (v6) and Ep-CAM are late events, since Ep-CAM is positive in lymph node metastasis in patients with loss of Ep-CAM at the invasive front (unpublished observations). This suggests that the loss we observed is not a genetic defect, but rather reflects the complex interactions of angiogenesis, adhesion, matrix degradation and inflammation, which take place during the process of invasion and metastasis.

We have demonstrated that Ep-CAM and CD44v6 provide prognostic information additional to the TNM stage in a large series of gastric cancer patients with well documented, prospectively collected data from a randomised trial (Bonenkamp *et al*, 1995, 1999). Both CD44v6 and Ep-CAM expression may be helpful in identifying behaviour of gastric adenocarcinoma. This information may be helpful in selecting patients suitable for surgery or for additional treatment pre- or postoperatively. However, additional studies are required to establish the place of these markers in clinical management of patients with gastric cancer.

## Figures and Tables

**Table 1:** TNM stage and marker expression related to overall survival

			**Overall survival**		
**Marker expression**	***n***	**(%)**	**5 year %**	**10 year %**	***P*-value (log-rank)**
*TNM-stage (n*=*285)*					
I	97	(34)	74	58	
II	61	(21)	36	26	
III	76	(27)	12	6	<0.001
IV	50	(18)	6	4	

*p53 (n*=*286)*					
0–10% positive	146	(51)	36	27	
⩾10% positive	140	(49)	35	25	0.85

*CD44v9 (n*=*286)*					
0–5% positive	148	(52)	37	27	
⩾5% positive	138	(48)	33	25	0.23

*VFF8 (v5) (n*=*282)*					
Negative	115	(41)	35	24	
Positive	167	(59)	36	27	0.84

*VFF18 (v6) (n*=*285)*					
0–25% positive	89	(31)	24	19	
26–100% positive	196	(69)	41	29	0.01

*VFF7 (v6) (n*=*284)*					
Negative	220	(78)	38	28	
Positive	64	(22)	27	19	0.11

*NEU (n*=*289)*					
Negative	263	(91)	35	26	
Positive	26	(9)	31	23	0.98

*E-Cadherin (n*=*281)*					
0–49% positive	146	(52)	34	25	
50– 100% positive	135	(48)	35	24	0.63

*Ep-CAM (n*=*280)*					
Negative	20	(7)	10	5	
1–99% positive	208	(74)	32	22	<0.001
100% positive	52	(19)	54	42	

**Table 2:** Association between TNM stage, VFF18 and Ep-CAM expression and survival in patients with a curative resection in intent

			**Overall survival**		***P*-value**
	***N***	**(%)**	**5 year %**	**10 year %**	**(log-rank)**
*TNM-stage (n*=*251)*					
I	97	(39)	73	58	
II	61	(25)	36	26	
III	77	(30)	12	6	
IV	16	(6)	19	13	<0.0001

*VFF18 (n*=*240)*					
0–25%	74	(31)	28	23	
26–100%	166	(69)	48	34	0.01

*Ep-CAM (n*=*236)*					
0	15	(6)	13	7	
1–99%	174	(74)	38	26	<0.001
100%	47	(20)	60	47	

**Table 3:** Cox's regression analysis applied on TNM stage, VFF18 and Ep-CAM expression (*n*=251)

	**RR**	**95% CI**	***P*-value**
*TNM stage*			
I+II	1.00	2.27–4.29	<0.001
III+IV	3.12	1.01–2.00	0.04

*VFF18*			
26–100%	1.00	0.99–2.31	0.05
0–25%	1.42	0.96–4.14	0.06

*Ep-CAM*			
100%	1.00		
1–99%	1.51		
0%	1.99		
